# Improving the reporting of randomised pilot and feasibility studies: a consort statement extension

**DOI:** 10.1186/1745-6215-16-S2-P16

**Published:** 2015-11-16

**Authors:** Sandra Eldridge, Christine Bond, Mike Campbell, Sally Hopewell, Lehana Thabane, Gill Lancaster, Claire Coleman

**Affiliations:** 1 Queen Mary University of London, London, UK; 2 University of Aberdeen, Aberdeen, UK; 3 Sheffield University, Sheffield, UK; 4 Oxford University, Oxford, UK; 5 McMaster University, Hamilton, Canada; 6 Lancaster University, Lancaster, UK

## 

Pilot and feasibility studies underpin much of current health related research, including randomised controlled trials. The number of reports in which authors describe their studies as pilot or feasibility studies is increasing and there is currently a lot of interest in this area. However, in spite of a number of papers that have recommended ways in which the reporting of these studies could be improved, reporting remains poor.

Using CONSORT endorsed methodology including a large Delphi study (n=93) and an international consensus meeting (n=26) we have developed a CONSORT extension for randomised pilot and feasibility studies. Much of the existing CONSORT statement for randomised controlled trials does apply to these types of study. However, sometimes the application of the items is different from that in RCTs designed to evaluate the effect of an intervention or therapy and some CONSORT items are not applicable or have needed some alteration.

We are currently writing the explanation and elaboration statement for this CONSORT extension. We will present the major issues in reporting these types of randomised studies and use examples to illustrate good and bad practice. This work is part of a larger programme of work on pilot and feasibility studies.

